# Genomic Selection for Antioxidant Production in a Panel of *Sorghum bicolor* and *S. bicolor × S. halepense* Lines

**DOI:** 10.3390/genes10110841

**Published:** 2019-10-24

**Authors:** Ephrem Habyarimana, Marco Lopez-Cruz

**Affiliations:** 1 CREA Research Center for Cereals and Industrial Crops, via di Corticella, 133-40128 Bologna, Italy; 2 Crop, Soil, and Microbial Sciences Department, Michigan State University, 1066 Bogue St, East Lansing, MI 42824, USA; malctony@hotmail.com

**Keywords:** *Sorghum bicolor*, *Sorghum halepense*, genomic prediction, genomic selection, antioxidants, genomic estimated breeding values, prediction accuracy

## Abstract

The purpose of this work was to assess the performance of four genomic selection (GS) models (GBLUP, BRR, Bayesian LASSO and BayesB) in 4 sorghum grain antioxidant traits (phenols, flavonoids, total antioxidant capacity and condensed tannins) using whole-genome SNP markers in a novel diversity panel of *Sorghum bicolor* lines and landraces and *S. bicolor × S. halepense* recombinant inbred lines. One key breeding problem modelled was predicting the performance in the antioxidant production of new and unphenotyped sorghum genotypes (validation set). The population was weakly structured (analysis of molecular variance, AMOVA R^2^ = 9%), showed a significant genetic diversity and expressed antioxidant traits with a good level of variability and high correlation. The *S. bicolor × S. halepense* lines outperformed *Sorghum bicolor* populations for all the antioxidants. The four GS models implemented in this work performed comparably across traits, with accuracy ranging from 0.49 to 0.58, and are considered high enough to sustain sorghum breeding for antioxidants production and allow important genetic gains per unit of time and cost. The results presented in this work are expected to contribute to GS implementation and the genetic improvement of sorghum grain antioxidants for different purposes, including the manufacture of health-promoting and specialty foods.

## 1. Introduction

Sorghum (*Sorghum bicolor* (L.) Moench) (2n = 2x = 20 chromosomes) is one of the world’s most important cereals grown for food, feed and biofuel [1]. Sorghum was traditionally a staple constituting a dominant portion of a standard diet for people in Africa and Asia, but it is becoming more popular worldwide, including in developed countries, for its uses in food industry to satisfy a rise in demand for specialty grains, especially those that are gluten-free and rich in health-promoting compounds [2]. This crop has interesting intrinsic properties including drought and heat resistance, adaptation to lower and higher latitudes, low input requirements and high energy balance, all of which makes sorghum suitable for use in current strategies to mitigate and adapt to climate change [3]. 

The DNA recombinant technology brought about several developments in molecular biology, including the next generation sequencing allowing rapid cost-effective whole-genome sequencing and genotyping. *S. bicolor* genome was first sequenced 10 years ago [4] with subsequent updates [5], has a physical size of 732.2 Megabases (Mb) with more than 34,000 annotated genes. Several investigations in genomic prediction and selection (GS) and genome-wide association studies (GWAS) in sorghum built upon this breakthrough [6,7,8,9,10]. The GWAS, also known as the linkage disequilibrium (LD) analysis, has improved the efficiency of marker assisted selection in comparison with the conventional linkage analysis. Quantitatively inherited traits, like antioxidants, are expected to include major and minor quantitative trait loci (QTL). The main feature of LD mapping is the possibility to detect and locate QTLs based on the strength of the correlation between mapped genetic markers and traits. This strategy relies on the decay of LD, initially present in a population at a rate determined by the genetic distance between loci and the number of generations since it arose. LD mapping offered greater precision in QTL location than family-based linkage analysis and lead to more efficient marker-assisted selection and gene discovery. Nonetheless, it showed its limits as it is unable to capture minor effects QTLs falling below the p-value threshold [11].

Genomic selection is a novel breeding paradigm that has been implemented in several crop species to address the shortcomings of the phenotypic selection [12,13,14,15,16,17,18] and the limitations of marker assisted selection (MAS) relying upon GWAS and linkage analyses. Genomic selectin is based on the infinitesimal model assuming that a trait value is a result of the linear combination of additive genetic and nongenetic sources and uses all available markers genome-wide to generate the predicted breeding value [19], effectively accounting for complex agronomic traits governed by many genetic components of predominantly small effect sizes [20]. On the other hand, marker assisted selection takes into account the effect of a small number of genetic markers that passed a significance threshold [21], which, according to the infinitesimal model, can only approximate the genomic underpinnings of a complex trait. MAS is therefore mostly effective for Mendelian traits controlled by a few genes of a large effect. 

The genomic selection equations trained with field phenotypes are used for selection purposes without field evaluation, meaning that genetic gain can be increased through increased population size and selection intensity. One of the attractive applications of GS in sorghum antioxidant breeding can be its implementation in early generations of recombinant lines or in the preliminary screening of germplasms to predict and select for or against the antioxidants’ concentration. The most interesting GS advantage relative to phenotypic, biochemical and proximal analyses is the possibility to account for the whole antioxidant QTLs and select superior strains early before seed setting. This is expected to shorten the time to cultivar development, reduce the number of lines to be field-tested and the cost of current breeding programs, help avoid committing resources on plant materials that are not expected to meet breeding and market standards, and ultimately bring about increased genetic gain per unit time and cost. 

It is now acknowledged that GS is well poised to be integrated and boost population genetics and quantitative genetics that remained for a long time without significantly benefitting from the DNA recombinant, next generation sequencing and genotyping technologies. Genomic selection is generally applied to facilitate recurrent selection (RS) schemes [22,23,24,24,25]. Recurrent selection (RS) is a cyclical breeding approach for the improvement of quantitative traits in breeding populations, and the production of novel gene combinations and increasing the frequency of favorable alleles [26,27]. Phenotypic recurrent selection (PRS) is implemented cyclically through the development of progeny (S0) by crossing selected parents, the evaluation of progeny (S1 or S2) to select new parents and recombining superior progeny to create the next cycle [28]. An accurate evaluation of progeny performance is limited by the lack of seed and resources for replicated trials. Genomic-assisted recurrent selection (GARS) would allow the prediction of S0 breeding values without the evaluation of S1 or S2 families in replicated trials. 

The efficiency of GS approach was evaluated in several crops, including sorghum. Further, some studies [8,9] investigated the importance of using pedigree and marker information to predict plant height, maturity, stay green and grain yield in sorghum testcross lines and evaluated the multi-traits genomic selection performance to improve sorghum drought adaptation and grain yield. Avelar de Oliveira et al. [10] implemented the Bayesian models Bayes A, Bayes B, Bayes Cπ, Bayesian LASSO (least absolute shrinkage and selection operator) and Bayesian RR (ridge regression) and rrBLUP to fit the random regression best linear unbiased predictor (RRBLUP) model and evaluated their efficiencies in phenotypic biomass traits including days to flowering, plant height, fresh and dry matter yield, fiber, cellulose, hemicellulose and lignin proportions in sorghum landraces and breeding lines. However, to the authors knowledge, no research work was conducted to evaluate the efficiency of GS in sorghum grain antioxidant concentration. This work was therefore designed to fill this gap.

## 2. Materials and Methods

### 2.1. Plant Materials

The sorghum materials used in this work consisted of 114 genotypes, of which 95 and 19 were, respectively, *Sorghum bicolor* (SB) and a mixture of recombinant inbred lines derived from *S. bicolor* × *S. halepense* (SBxSH) controlled crosses at different levels of filial progeny. These populations were described in our previous works [1,29]. *Sorghum bicolor* genotypes were annual sorghum selections derived from landraces from Africa and Asia, while the *S. bicolor* × *S. halepense* advanced recombinant lines were selections from annual/perennial (A/P) crosses and A/P backcrosses to annual recurrent parents (A*2/P; BC1), perennial/perennial (P/P) and annual/perennial//perennial (A/P//P) crosses. The annual parents were induced tetraploids (2n = 40), standard diploid (2n = 20), genetic male-sterile and cytoplasmic-genetic male-sterile inbred *S. bicolor* lines. The perennial parents were a *S. halepense* plant and tetraploid lines obtained by crossing induced tetraploid sorghum plants with *S. halepense*. These 114 genotypes were evaluated in open-field trials in 2014 and 2015 at CREA Research Center for Cereal and Industrial Crops, in the experimental station of Anzola (Bologna, Italy), using an augmented randomized complete block design with 6 controls (checks) and 6 blocks [30]. The seeds were hand-harvested a few days after the plants reached the physiological maturity as indicated by the appearance of the black layer (hilum) in the caryopses at the base of the panicle. 

### 2.2. Determination of Antioxidants Concentration and Statistical Inferences

To analyze total phenols, tannins, flavonoids and antioxidant capacity (TAC), a 10 g sample from each genotype was ground using a Cyclotec Udy Mill (sieve: 0.5 mm). The moisture in the sample was determined after they were oven-dried overnight at 105 °C and antioxidants and TAC were analyzed in duplicate using 100 mg of each sample as described in our previous work [2,31]. For the phenolic compounds, the absorbance of samples was measured at 750 nm and expressed as gallic acid equivalents (gGAEkg^−1^ dry mass basis). For condensed tannins and total flavonoids assays, the absorbances were measured at 500 nm and 510 nm, respectively, and expressed as μg CE (catechin equivalents) g^−1^ dry mass basis. The TAC was determined using the 2,20-azino-bis/3-ethylbenzthiazo- line-6-sulphonic acid (ABTS) assay and expressed as mmol TE (Trolox equivalents) kg^−1^ dry basis. Statistical inferences were performed using R, a statistical computing language and environment [32]. 

To evaluate the genotypic variability for the evaluated traits, the Bayesian regression models in R was used using the probabilistic programming language Stan, implementing Hamiltonian Monte Carlo [33,34] and its extension, the no-u-turn sampler (NUTS) [35]. The model was well described in [36]. These algorithms converge much more quickly relative to other commonly used MCMC algorithms, like the Metropolis Hasting and Gibbs samplers [35]. The core model implemented in this work was the prediction of the response y by predicting all parameters $\theta_{p}$ of the response distribution D, also known as model family [36]. In this work, the effects of the genotypes on the variability of the traits of interest were evaluated by solving the following model $y_{i}\sim D\left(\theta_{1i},\theta_{2i},\ldots ,\theta_{pi}\right)$ for *i*th observation, with $\theta_{pi}=f_{p}\left(\eta_{pi}\right)$. The models were implemented with one random intercept and no covariates, and therefore: η=Xβ+Zu, where β and u are the respective coefficients at the population level and group level, and X, Z are the corresponding design matrices. The default rules were applied to choose hyperparameters. For each trait, the models were fitted using 4 chains, each with 50,000 iterations of which the first 10,000 were warmup (burn-in) to calibrate the sampler, leading to a total of 160,000 posterior samples upon which our analyses were based. Genotypic variability was measured using the mean (estimate) and the standard deviation (estimate error) of the posterior distribution as well as two-sided 95% credible intervals (l-95% CI and u-95% CI) based on quantiles.

Variance components and trait broad-sense heritability (repeatability, hereafter referred to as heritability) [37,38,39] were estimated by fitting the linear mixed model equation $y_{ij}=\mu +g_{i}+e_{ij}$ for i=1,…,s genotypes, $j=1,\ldots ,n_{i}$ replicates (year) for genotype i, $y_{ij}$ is the response variable for genotype i in replicate (year) j; it was assumed $g_{i}\sim N\left(0,\sigma_{g}^{2}\right)$ and $e_{i}\sim N\left(0,\sigma_{e}^{2}\right)$ [40]. The yearly adjusted means were used and the model was fitted with restricted maximum likelihood using the R package lme4 [32]. The heritability was derived through the formula $\sigma_{g}^{2}/\left(\sigma_{g}^{2}+\sigma_{e}^{2}/n_{r}\right)$ [10,41] where $\sigma_{g}^{2}$, $\sigma_{e}^{2}$ and $n_{r}$ are the genetic (genotypic) variance, residual variance and the number of replications (years), respectively. 

### 2.3. DNA Extraction

In samples grown in summer, the genomic DNA was isolated from young (3 weeks old) seedlings grown in pots under the glasshouse. Five to twenty sorghum seeds per sample were sown in peat, watered and were treated with a fungicide (Ortiva, Syngenta, 1 mL L^−1^) and an insecticide (Confidor, Bayer, 0.75 mL L^−1^) for plant protection purposes. For the samples grown in winter, the seeds were treated with a seed-coating fungicide (Celest, Syngenta, 4 mL L^−1^ in water) and put to germinate on wet filter paper within petri dishes laid in the incubator Venticell 111 (MMM group) at 25 °C for 4–6 days. Then, 1–3 healthy plantlets (nearly 10–30 cm tall) per pot or 3–5 sprouts per petri dish were collected for DNA extraction. DNA was extracted using the GeneJET Plant Genomic DNA Purification Kit (ThermoFisher Scientific, Waltham, MA, USA), following the manufacturer’s instructions. The DNA concentration and purity were evaluated by a Tecan Infinite M200Pro spectrophotometer (Tecan Group Ltd., Männedorf, Switzerland), while DNA integrity was checked through 1% agarose gel electrophoresis containing 10 µL L^−1^ GelRed (Biotium) as fluorescent dye. For each DNA sample, an aliquot of 60 µL at a concentration ≥ 10 ng µL^−1^ was used for the subsequent GBS SNP genotyping. 

### 2.4. Whole-Genome GBS SNP Genotyping and Population Structure

Whole-genome genotyping-by-sequencing (GBS) strategy was carried out on the panel of 114 sorghum lines previously phenotyped for antioxidants production. For glasshouse-related logistical reasons, the GBS was run in two batches of 184 and 196 individuals, corresponding to the two separate sample deliveries. The ApeKI, a methylation-sensitive restriction enzyme, was used for library preparation and GBS was carried out on an Illumina HiSeq X Ten platform by BGI Hong Kong Company Limited. The sequencing reads were aligned to the sorghum reference genome (Sorghum_bicolor NCBIv3) to enable variants discovery. The two batches yielded two respective matrices of 933,020 and 919,485 markers that were delivered in separate variant call format (VCF) files stored in a solid-state disk. The two VCF files were subsequently merged into a single matrix using VCFtools [42] resulting in a total of 1,252,091 loci. The marker quality control criteria were applied to the merged dataset considering only genotyped and phenotyped samples. The filters were implemented in VCF tools to restrict the dataset to high quality standards including biallelic SNPs only, minor allele frequency (MAF) ≥ 0.05, site quality or the Phred-scaled probability that reference/alternative alleles polymorphism exists at a given site given the sequencing data Q ≥ 40 (i.e., ≥99.99% base call accuracy) and missing genotypes (NA) ≤ 20%. The quality-controlled marker data consisted of 61,976 high-quality SNPs which were subsequently used in this work for genomic prediction and selection analyses. 

The density of these high-quality SNP markers was visualized using CMplot in R. The population structure was investigated based on molecular marker information using a Bayesian model-based clustering [43], neighbor-joining algorithm and the analysis of molecular variance (AMOVA). The neighbor-joining cluster analysis was performed using Euclidean distances in the ape package implemented in the R program [32]. In the Bayesian model-based clustering, shared allele frequencies and an admixture model were applied to investigate the number (K) of populations. The initial burning period was set to 500 followed by 50,000 MCMC (Markov chain Monte Carlo) iterations for each run. The optimal K was determined following the guidelines provided by Evanno et al. [44]. Further, analysis of molecular variance was performed in R as the hierarchical analysis of molecular variance as described in [45]. The clusters were considered as the source of variation and the number of permutations was set to 1000. 

### 2.5. GS Models and Prediction Accuracy

Four parametric GS models were implemented in this work and included genomic best linear unbiased prediction (GBLUP), Bayesian ridge regression (BRR), Bayesian LASSO (BL) and Bayes B. The relevance of these algorithms in genomic prediction and selection was amply described in previous works (e.g., [39,46]). However, Bayesian ridge regression is equivalent to GBLUP [47] but with variance parameters estimated Bayesianly [48]. The models were selected as they differ in the assumptions of the distribution of marker effects and can therefore offer the possibility to account for different models of genetic variation and accommodate diverse modes of inheritance in different traits. For example, GBLUP predicts breeding values by borrowing information among relatives through their kinship [49] calculated from genomic data. However, genome-wide regression models (BRR, BL, Bayes B) rely on the infinitesimal model of quantitative genetics, assuming that the genetic control of complex traits is equally distributed across many (infinite) loci with small effects [19,50]. The genomic estimated breeding values (GEBVs) for the GBLUP method are obtained from the best linear unbiased predictions (BLUPs) of the random additive effect. The method BRR is a Bayesian version of ridge regression, in which the marker effects are normally distributed (i.e., Gaussian prior), have identical variance and are shrunk toward zero. Therefore, the genomic best linear unbiased prediction and BRR methods are appropriate for traits controlled by a large number of genes with small effects i.e., the infinitesimal model. The BL method is expected to be best suited to the finite loci model with stronger shrinkage of regression coefficients that are close to zero and less shrinkage of those with large absolute values [51,52]. In contrast, the Bayes B approach allows the marker loci to explain the different amounts of variation, with only a small number of loci having a non-zero effect and many loci having no effect, and can therefore be best suited to assumptions made by the QTL model which is based on a small number of genes having a large effect and many genes having a null effect [52]. 

Descriptively, the genomic selection models assumed that the phenotypic outcomes $y_{i}\left(i=1,2,3,\ldots ,n\right)$ were the sum of a genetic component represented by marker covariates $x_{ij}\left(j=1,2,3,\ldots ,p\right)$ and a non-genetic component (residual, $\varepsilon_{i}$) as translated in the linear regression model of the form $y_{i}=\mu +\overset{p}{\underset{j=1}{\sum }}x_{ij}\beta_{j}+\varepsilon_{i}$, where μ is the intercept, $x_{ij}$ the genotype of the *i*th individual at the *j*th marker, $\beta_{j}$ is the corresponding coefficient of regression of $y_{j}$ on the *j*th marker covariate. The residual was assumed to follow normal distribution with constant variance i.e., $\varepsilon \sim N\left(0,\sigma_{\varepsilon }^{2}I\right)$. Since p was much larger than n, the models were penalized and regularized as suggested by [46]. To evaluate the GS model performance, the Monte Carlo (repeated hold-out) cross-validation approach [53,54] was applied using 70% and 30%, respectively, as training and validation sets. In a standard hold-out cross-validation, the data is randomly divided into two subsets: a training and a test (validation) set. The test set represents new, unseen data to the model. To obtain a more robust performance estimate that was less variant to how the data was split into training and test sets, the holdout method was repeated 50 times with different random seeds and the average performance was computed over these 50 repetitions. The repeated hold-out procedure provides a better estimate of how well our model may perform on a random test set, compared to the standard holdout validation method [54]. In addition, it provides information about the model’s stability as to how the model, produced by a learning algorithm, changes with different training set splits. 

The model parameters were estimated in the training set and the models validated in the testing set. The performance of the models (accuracy) was measured as the Pearson correlation coefficient (r) between the phenotypic data (adjusted means) and the genomic estimated breeding values of the individuals in the testing set. The models were implemented using R software, version 3.5.3 (R Core Team, 2013) and the package BGLR [46] by applying default rules for choosing hyperparameters. The Gibbs sampler was used and our analyses was based on 30,000 samples from the posterior distribution obtained after the first 5000 iterations were discarded as burn-in [1]. The diagrams and statistical inferences used to represent the GS models’ outcomes were made using appropriate routines called from the R software. 

## 3. Results

### 3.1. Phenotypic Performance and Diversity

**Figure 1:**
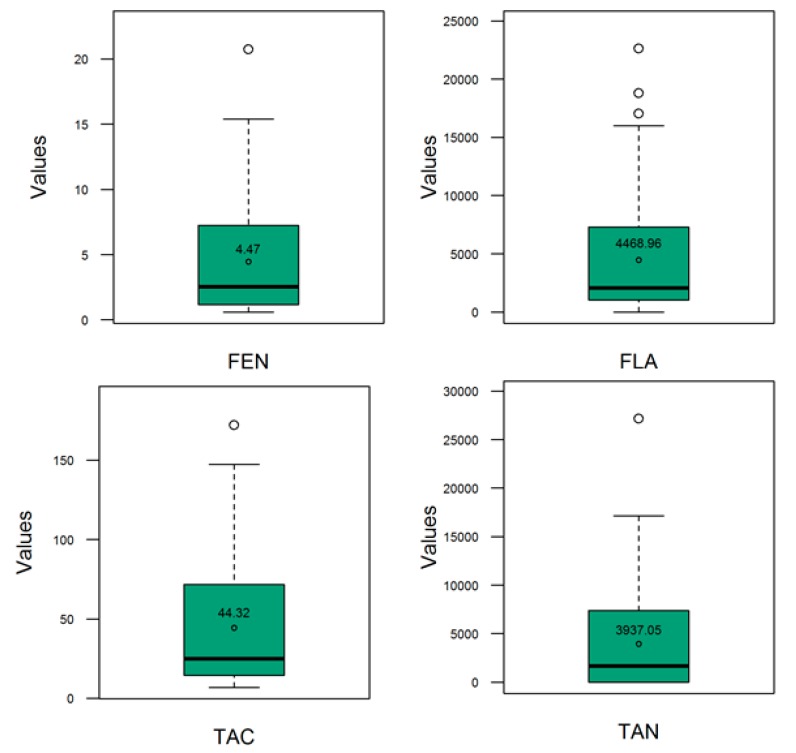
Distribution (boxplot) of polyphenol (FEN), flavonoids (FLA), condensed tannins (TAN), and total antioxidant concentrations. The open dot and the numbers inside the green rectangles (interquartile ranges) represent, respectively, the position of mean and the mean of the trait of intertest. The horizontal bar inside the green rectangles represents the median of the trait of interest. Refer to text for the description of the traits.

The descriptive statistics of the contents of the antioxidants measured in the sorghum populations evaluated in this work, are depicted graphically using quartiles (Figure 1). A wide range in the measured traits was observed and reflected the existence of a good phenotypic variability. In the entire sorghum panel, the mean of the total antioxidant capacity (TAC), phenols (FEN), tannins (TAN), and flavonoids (FLA) was, respectively, 43.31 mmol TE kg^−^^1^ dm^−1^, 4.36 g GAE kg^−1^ dm^−1^, 5349.23 μg CE g^−1^ dm^−1^ and 5300.49 μg CE g^−1^ dm^−1^, while the range was 6.88𠄲172.02 mmol TE kg^−1^ dm^−1^, 0.60𠄲20.73 g GAE kg^−1^ dm^−1^, 66.89𠄲27,138.56 μg CE g^−1^ dm^−1^ and 207.62𠄲22,606.17 μg CE g^−1^ dm^−1^, respectively. 

The output of the Bayesian regression models is summarized in Table 1. The estimate of the effective sample size (ESS) was satisfactory across traits, with bulk effective sample size (ESS) and tail ESS > 400. The ESS is the number of independent samples from the posterior distribution that would be expected to yield the same standard error of the posterior mean as is obtained from the dependent samples returned by the MCMC algorithm. The threshold in the Bayesian regression model using Stan is 100 times the number of MCMC chains run [36]. The bulk ESS and tail ESS measure, respectively, the reliability of posterior means and medians, and posterior variances. The Rhat (potential scale reduction factor on split chains; refer to Table 1 for further descriptions) values were lower than 1.1, meaning that the generated Markov chains converged [55] to the respective posterior (stationary) distributions of interest. On the other hand, none of the 95% credible intervals contained zero (the parameter value specified in the null hypothesis), implying that the evaluated population parameters in Table 1 were significantly different from zero at the 0.05 level. The estimates of standard deviation of the random intercepts (measures of genotypic effects on the response variables) were higher in FLA (4461.34) and TAN (3935.62), followed by TAC (37.93) and FEN (4.26), meaning that there was individual genotype-level variability in the traits evaluated in this work. The intercepts were 5276.13, 4.33, 43.21, and 5320.1, respectively, for FLA, FEN, TAC, and TAN. The residual (unexplained) standard deviation (sigma) was 405.25, 253.67, 2.71, and 0.22, respectively, in FLA, TAN, TAC, and FEN and represented 9% or less than the observed standard deviation of the genotypic effects across traits. 

**Figure 2:**
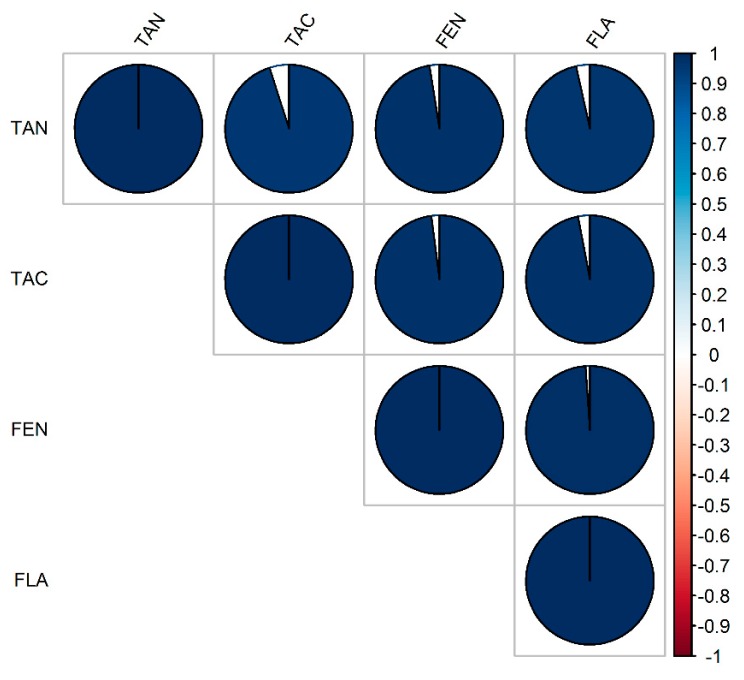
Pearson correlation coefficients among the evaluated traits. FEN, FLA, TAC, TAN, respectively, polyphenols, flavonoids, total antioxidant capacity, and condensed tannins. The filled-in areas of the circles show the absolute value of corresponding correlation coefficients. The scale on the right-hand side is colored from red (negative correlation) to blue (positive correlation); with the intensity of color scaled 0–100% in proportion to the magnitude of the correlation.

The broad-sense heritability was very high with values greater than 0.99 (data not shown) in all the traits. The pairwise Pearson correlation coefficients among the four antioxidant traits (Figure 2) were positive and very high (r > 0.90) and ranged from 0.92 (TAN-TAC) to 0.98 (TAC-FEN). 

### 3.2. GBS SNP Whole-Genome Genotyping

**Figure 3:**
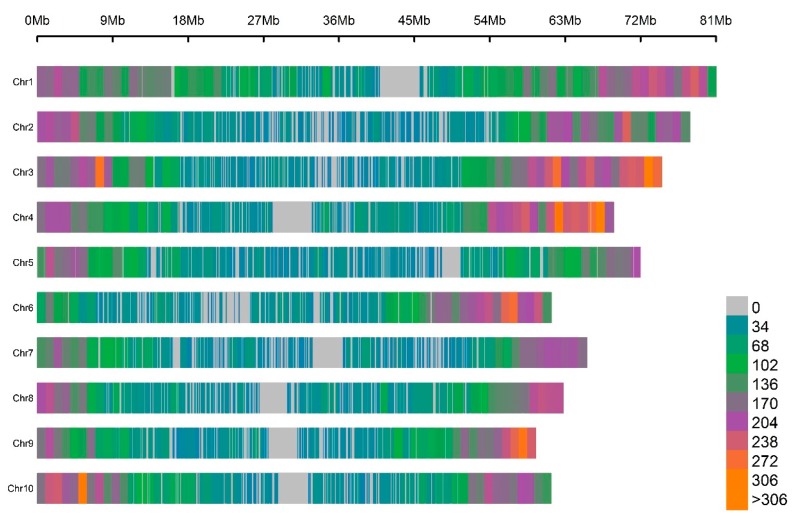
Chromosome-wise density (number of SNPs called) of genotyping-by-sequencing (GBS) SNP markers discovered in the population of 114 sorghum materials evaluated in this work. The color-coded scale bar to the left indicates the respective number of SNPs within 1 Mb window size.

The next generation sequencing and genotyping of the panel of 114 sorghum lines evaluated in this work yielded an unfiltered matrix of 1,252,091 SNP marker loci which, after quality control, resulted in a final working matrix consisting of 61,976 high-quality SNPs. The physical maps of the 61,976 discovered high-quality markers covered the 10 chromosomes (Chr) of Sorghum bicolor (Figure 3). The relative physical lengths (in Mb) of the 10 chromosomes (linkage disequilibrium groups) was, in decreasing order, Chr1 (81) > Chr2 (78) > Chr3 (74) > Chr5 (72) > Chr4 (69) > Chr7 (66) > Chr8 (63) > Chr6 (61) > Chr10 (61) > Chr9 (59). The density of the discovered SNPs was higher in the chromosomal arms and increased from the centromeres to the telomeres. The SNP markers were scarce or absent in the centromeric regions. 

### 3.3. Population Structure

**Figure 4:**
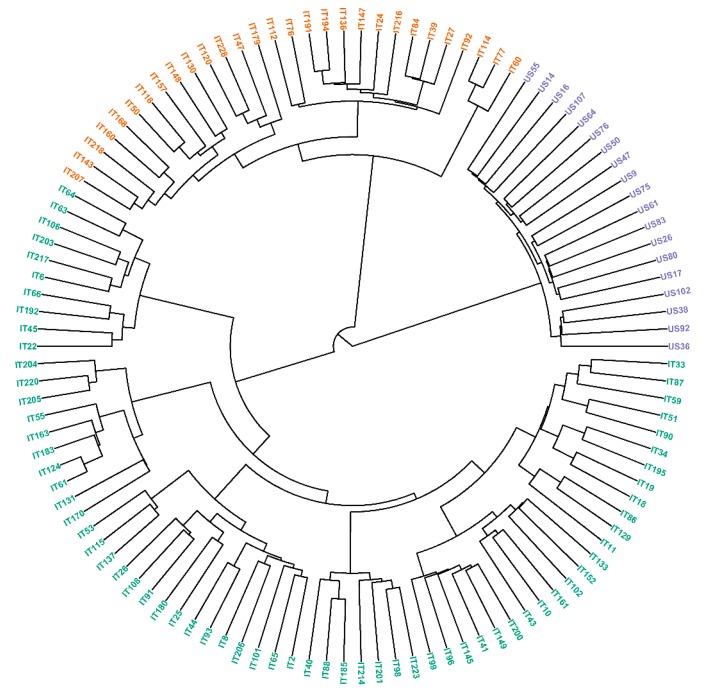
Neighbor-joining clustering of the population of the 114 sorghum lines evaluated in this work. Cluster A (slightly desaturated blue color), Cluster B (vivid orange color), Cluster C (dark-cyan color).

**Figure 5:**
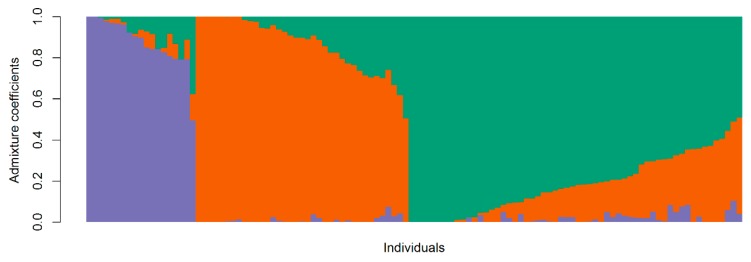
Model-based structure of the population of the 114 sorghum lines evaluated in this work. Cluster A (slightly desaturated blue color), Cluster B (vivid orange color), Cluster C (dark-cyan color).

The 114 sorghum genotypes evaluated in this work clustered in three main groups using neighbor joining (Figure 4) and model-based structure (Figure 5) algorithms. Cluster A (slightly desaturated blue color) included the 19 *Sorghum bicolor* × *S. halepense* recombinant lines, cluster B (vivid orange color) included 37 Sorghum bicolor breeding lines developed mainly from Southern and Eastern African *Sorghum bicolor* lines and landraces, while in cluster C (dark-cyan color) 58 *Sorghum bicolor* breeding lines developed mainly from Asian Sorghum bicolor lines and landraces were grouped. As shown by the neighbor joining algorithm, these main clusters included several sub-clusters and the model-based structure algorithm showed that most of the lines included different levels of admixture coefficients values. The analysis of molecular variance (AMOVA) resulted in highly significant effects (*p* = 0.001) of the clusters on the molecular variance observed in the GBS SNP dataset (Table 2). The coefficient of determination (R^2^) was calculated to assess how well the three-cluster model explained the variability in the data set. In this work, R^2^ was low (9%), meaning that the AMOVA model based on the three clusters did not fully account for the diversity that existed in the population.

### 3.4. Genomic Prediction of Genetic Merit

**Figure 6:**
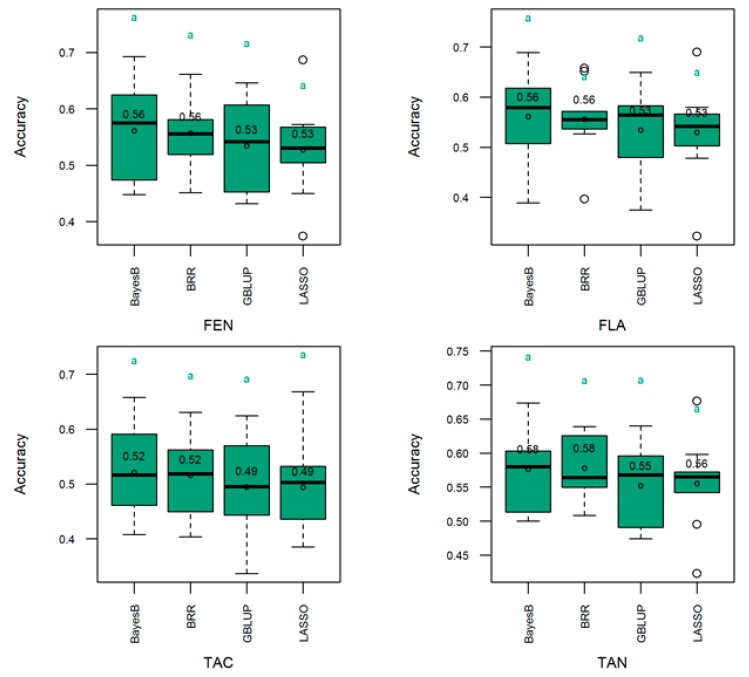
Distribution (boxplot) of GS models validated accuracy in external sample (not used during model training) of 34 (30% of the total population) sorghum lines. FEN, FLA, TAC, TAN, respectively, polyphenols, flavonoids, total antioxidant capacity, and condensed tannins. Traits means are included within the boxplot. Trait means with same letter are not significantly different at the 5% level using the Tukey’s HSD (honestly significant difference) test. Refer to text for the description of the GS models.

The main feature of the genomic selection approach is represented by the possibility to compute the genomic estimated breeding value (also known as genetic merit) upon which superior strains are selected without conventional trials and phenotyping. Four GS models were implemented in this work to evaluate their performance in predicting the genetic merit for the production of polyphenols, flavonoids, total antioxidants and condensed tannins in 114 sorghum genotypes. The predicting ability as represented by the respective GS model accuracy is presented in Figure 6. GBLUP, BRR, BL and BayesB models performed comparably across traits. The prediction accuracy ranges were 0.53–0.56 for polyphenols and flavonoids, 0.49–0.52 for the total antioxidant capacity and 0.55–0.58 for condensed tannins. 

## 4. Discussion

Compared to other grains, sorghum contains high levels of antioxidants, which can help prevent cell and DNA damage and play a role in degenerative disease prevention [56]. Sorghum breeding programs rely heavily on unreliable phenotypic selection for the contents of antioxidants in the grains, using genomic breeding values estimated with genomic selection models can simplify and expedite breeding for antioxidant in sorghum. Genomic selection was recommended for use particularly in quantitatively inherited traits and traits with low heritability or which are hard to measure [11,39]. Although the antioxidant traits evaluated in this study exhibited a high value of broad-sense heritability, Genomic selection can nonetheless be recommended for antioxidant breeding in sorghum for several reasons. First, the use of grain color as a proxy to antioxidant concentration is complicated by the necessity of having a variety of information including pericarp thickness, pigmented testa, spreader genes and endosperm appearance, which are naturally correlated with the production of sorghums with increased phenols and antioxidant activity levels [57,58]. Second, there are many other compounds in sorghum grain that contribute to antioxidant activity, including vitamin E and carotenoids, most of which are hard to measure or are associated with minor effects QTLs that cannot be detected for use in marker assisted selection [7]. Third, for most sorghum antioxidants, an analysis through wet chemistry is more costly than GBS SNP genotyping, whereas the cost of high-throughput analyses (e.g., near-infrared spectroscopy, NIRS) is currently comparable to GBS SNP genotyping. Nonetheless, even in cases of comparable costs, GBS SNP genotyping to select superior untested and unphenotyped individuals offers unique advantages, such as the possibility to operate selection early at the seedling stage without waiting until seed setting or harvest, which can drastically expedite and reduce the cost of breeding programs. 

This work aimed at conducting GS for sorghum grain antioxidants using SNP markers in a novel diversity panel consisting of a combination of *Sorghum bicolor* landraces and a progeny derived from *S. bicolor* × *S. halepense* (SBxSH) hybridizations. To the best of the authors’ knowledge, it is the first time GS approach is performed to predict antioxidants in sorghum grains. The different metrics used in this work highlighted the important diversity that existed in the evaluated sorghum materials. Using a quantile approach and the Bayesian regression model with Stan revealed a good level of phenotypic variability that was significantly explained by the differences between the evaluated sorghum lines (Figure 1, Table 1). Neighbor joining and the model-based structure identified three main clusters and the latter algorithm showed the existence of genetic admixtures. On the other hand, the analysis of molecular variance showed the existence of a statistically significant but weak (R^2^ = 9%) genetic differentiation. The lower coefficient of determination (R^2^) meant that much of the variance and the structure observed in the population could be attributed to other sources, such as differences between individuals within clusters and/or between individuals, and admixture, all of which can be associated with different levels of pairwise kinships. 

The observed diversity is associated with the intrinsic genetic properties of the plant materials used in this work. The landraces and, particularly, the *S. halepense* genome constitute a good source of antioxidants [59]. In SBxSH combinations, the wild genome of *S. halepense* represents an untapped reservoir and a good source of genetic diversity that can be used in conventional and molecular breeding. On the other hand, the *S. bicolor* genotypes were derived from African and Asian landraces and are expected to harbor a high level of genetic diversity for breeding purposes inasmuch as Africa and Asia represent, respectively, the primary and secondary sorghum centers of diversity [29]. Furthermore, the SBxSH lines and *S. bicolor* landraces evaluated have a long history spanning, respectively, more than 6 and 20 years in our breeding program. They have accumulated cycles of meiotic and recombination events that brought about genetic diversity, which qualify the sample size used herein for investigations in genomic prediction [60,61,62]. 

The broad-sense heritability of the traits (FEN, FLA, TAC, TAN) evaluated in this work was very high, indicating that the phenotypic variations of the measured antioxidant metrics are mainly affected by genetic factors and therefore this panel can be used for genomic prediction and selection. On the other hand, the pairwise correlation among the four antioxidant traits (Figure 2) was positively very high implying that the proportion of variance shared by the traits evaluated was mostly due to genetic causes. It can be inferred that the observed high correlation between antioxidant components and the TAC is an indication that the antioxidant components evaluated in this work were a major source of antioxidant activity in the sorghum grain. A perfect correlation between traits implies that genetic influences on the traits of interest are identical and this situation can imply the existence of either pleiotropy (causal overlap), linkage disequilibrium, or ascertainment bias as related to sampling bias. 

### 4.1. Contribution of S. halepense to Antioxidant Improvement in Sorghum

The purpose of this work was to conduct GS of sorghum grain antioxidants using SNPs in a novel diversity panel of *Sorghum bicolor* landraces and *S. bicolor × S. halepense* recombinant inbred lines. To the best of the authors’ knowledge, it is the first time GS prediction modelling is implemented in sorghum antioxidants. *Sorghum halepense* is mostly used in hybridizations with domesticated *S. bicolor* with the aim of introgressing perenniality into the genetic background of *S. bicolor* but, it can also be used for genetic improvement of several other traits [63], such as antioxidant properties in sorghum grain. This is particularly important as wild sorghums generally show a higher antioxidant content compared to domesticated ones [59] in which these compounds were artificially selected against. Indeed, in this work, *S. halepense* × *S. bicolor* lines statistically (at the 0.05 probability level) outperformed *S. bicolor* populations in terms of total antioxidant capacity (TAC, 59.16 versus 40.17 mmol TE kg^−1^ dm^−1^) and the contents of tannins (7358.57 versus 3053.25 μg CE g^−1^ dm^−1^), phenols (6.56 versus 3.93 g GAE kg^−1^ dm^−1^) and flavonoids (6738.39 versus 3869.66 μg CE g^−1^ dm^−1^). The *S. halepense* × *S. bicolor* lines evaluated in this work can therefore be considered as a good source of antioxidants variability in addition to the possibility of using them to breed for perennial grain sorghum fortified with antioxidants. Nonetheless, the genomic selection for grain quality and production in a population derived from the hybridization *Sorghum bicolor × Sorghum halepense* is expected to be challenging particularly due to the necessity to eliminate negative wild-related traits such as loose panicle, tiny seeds and seed chattering. The different breeding strategies can be applied, including combining different traits in an index of selection with appropriate weights or applying a breeding scheme (e.g., tandem selection) to cull the undesired traits first, before proceeding with breeding for antioxidant production. 

### 4.2. Genomic Selection Model Performance

Marker density resulted in a physical map length comparable to the sizes of the chromosomes of the sorghum reference genome (Sorghum_bicolor NCBIv3) [5]. The density of the discovered SNPs was lower in the centromeric regions, which is consistent with these constitutive heterochromatin regions being poor in expressed genes, and with the results in [64] showing that GBS SNPs were preferentially located either in or near gene-rich regions. The four GS models implemented in this work performed comparably across traits (Figure 6) with GS accuracy ranging from 0.49 to 0.58 (Figure 6). This can be explained by the high pairwise correlation between the evaluated traits which can result from the controlling pleiotropic genetic factors and/or blocks of genetic factors that are in linkage disequilibrium among themselves and with the traits of interest. In such situations, the GS models captured molecular information shared among traits, resulting in comparable prediction performance. The genomic selection investigations reported herein were based on phenotypic data collected from two open field trials conducted over two years. The two-year trials are a threshold standard in agricultural research. Several studies, e.g., [6,7,65] similar to ours, reported genomic prediction findings based on one-year trials. Sorghum antioxidants concentration is a highly heritable trait. Indeed, heritability in this work was above 0.9, meaning that the environmental noise for these qualitative traits is negligible and could not significantly affect the statistical inferences on which our findings were based. 

Three major factors that affect the GS accuracy are the training set size, marker density and population structure [66]. In this work, the training set size ($n_{trn}=$ 80) was meaningful and it was higher or comparable to the sizes experimented in previous works (e.g., [67]). In addition, the GS accuracy was consistently moderately high (≥ 0.5) across traits implying that the observed weak population structure did not impact the performance of the GS models. Regarding the marker density, the number of the markers used in this work covered the entire length of the sorghum genome (Figure 3) and was higher than or comparable with several previous works (e.g., [8,9,39]). 

The available empirical evidence for GS efficiency in plant breeding is set to 0.5, the baseline for GS prediction accuracy in plant breeding [14,67]. In his recent work on the hexaploid bread wheat, [38] demonstrated that GS accuracy as low as 0.2 can allow substantial within-generation grain yield improvement. Therefore, the GS model performances obtained in the present study are high enough to sustain sorghum breeding for antioxidants production and allow important genetic gains per unit of time and cost. In addition to the accuracy, the importance of the GS strategy is also evaluated using other criteria, such as the possibility GS offers to shorten the breeding cycle with interesting economic returns [67] due to intercrosses driven by genetic predictions, and in the case of antioxidants, GS offers the possibility of selecting for or against this trait early (e.g., at the seedling stage) without waiting for seed setting or harvest. The GS equations developed in this work can be directly used in sorghum breeding programs. The GS results presented herein and experimental designs used in this work can be implemented in antioxidants genetic investigations and in breeding programs to qualitatively and quantitatively improve the antioxidant production for different purposes including the manufacture of health-promoting and specialty foods. 

## Figures and Tables

**Table 1:** Mean (estimate) and standard deviation (estimated error) of the posterior distributions, and the quantile-based confidence intervals.

Traits	Parameters	Estimate	Est. Error	l–95% CI	u–95% CI	Rhat	Bulk_ESS	Tail_ESS ^1^
FLA	SD.G (intercept)	4461.34	291.64	3932.56	5070.77	1	8469	18871
	Intercept	5276.13	415.54	4448.66	6083.73	1	2793	5094
	Sigma	405.25	26.3	357.83	460.87	1	113,402	190,944
FEN	SD.G (intercept)	4.26	0.28	3.75	4.86	1	2137	4694
	Intercept	4.33	0.39	3.55	5.1	1.01	466	1355
	Sigma	0.22	0.01	0.19	0.25	1	45,376	84,449
TAC	SD.G (intercept)	37.93	2.48	33.45	43.24	1	1787	3107
	Intercept	43.21	3.56	36.33	50.26	1.01	599	1491
	Sigma	2.71	0.18	2.4	3.08	1	32,486	56,955
TAN	SD.G (intercept)	3935.62	256.08	3469.61	4468.41	1	1555	3174
	Intercept	5320.1	358.8	4630.73	6044.63	1	501	887
	Sigma	253.67	16.37	224.16	288.35	1	23,079	44,792

^1^ l–95% CI, u–95% CI: lower and upper limits of the 95% credible interval; Rhat: potential scale reduction factor on split chains (at convergence, Rhat = 1), ESS: effective sample size, Sigma: residual standard deviation, SD.G (a measure of genotypic effects): standard deviation of the intercept at the individual genotype level. FEN, FLA, TAC, TAN, respectively, polyphenols, flavonoids, total antioxidant capacity, and condensed tannins.

**Table 2:** Analysis of molecular variance (AMOVA).

Source of Variation	Degrees of Freedom	Sums of Squares	Mean Squares	F Value	R^2^	*p* Value
Clusters	2	37,773.14	37,773.14	11.47	0.09	0.001
Residuals	111	368,746.64	3292.38	NA	0.91	NA
Total	113	406,519.77	NA	NA	1	NA

## References

[1] Habyarimana E., Lorenzoni C., Marudelli M., Redaelli R., Amaducci S. (2016). A meta-analysis of bioenergy conversion relevant traits in sorghum landraces, lines and hybrids in the Mediterranean region. Ind. Crops Prod..

[2] Alfieri M., Balconi C., Cabassi G., Habyarimana E., Redaelli R. (2017). Antioxidant activity in a set of sorghum landraces and breeding lines. Maydica.

[3] Habyarimana E., Bonardi P., Laureti D., Di Bari V., Cosentino S., Lorenzoni C. (2004). Multilocational evaluation of biomass sorghum hybrids under two stand densities and variable water supply in Italy. Ind. Crops Prod..

[4] Paterson A.H., Bowers J.E., Bruggmann R., Dubchak I., Grimwood J., Gundlach H., Haberer G., Hellsten U., Mitros T., Poliakov A. (2009). The Sorghum bicolor genome and the diversification of grasses. Nature.

[5] Mccormick R., Truong S., Sreedasyam A., Jenkins J., Shu S., Sims D., Kennedy M., Amirebrahimi M., Weers B., McKinley B. (2018). The Sorghum bicolor reference genome: Improved assembly, gene annotations, a transcriptome atlas, and signatures of genome organization. Plant J..

[6] Rhodes D.H., Hoffmann L., Rooney W.L., Ramu P., Morris G.P., Kresovich S. (2014). Genome-wide association study of grain polyphenol concentrations in global sorghum [Sorghum bicolor (L.) Moench] germplasm. J. Agric. Food Chem..

[7] Rhodes D., Gadgil P., Perumal R., Tesso T., Herald T.J. (2017). Natural Variation and Genome-Wide Association Study of Antioxidants in a Diverse Sorghum Collection. Cereal Chem. J..

[8] Velazco J.G., Malosetti M., Hunt C.H., Mace E.S., Jordan D.R., van Eeuwijk F.A. (2019). Combining pedigree and genomic information to improve prediction quality: an example in sorghum. Theor. Appl. Genet..

[9] Velazco J.G., Jordan D.R., Mace E.S., Hunt C.H., Malosetti M., van Eeuwijk F.A. (2019). Genomic Prediction of Grain Yield and Drought-Adaptation Capacity in Sorghum Is Enhanced by Multi-Trait Analysis. Front. Plant Sci..

[10] De Oliveira A.A., Pastina M.M., de Souza V.F., da Costa Parrella R.A., Noda R.W., Simeone M.L.F., Schaffert R.E., de Magalhães J.V., Damasceno C.M.B., Margarido G.R.A. (2018). Genomic prediction applied to high-biomass sorghum for bioenergy production. Mol. Breed..

[11] Muleta K.T., Pressoir G., Morris G.P. (2019). Optimizing Genomic Selection for a Sorghum Breeding Program in Haiti: A Simulation Study. G3 Genes Genomes Genet..

[12] Habier D., Fernando R.L., Dekkers J.C.M. (2009). Genomic Selection Using Low-Density Marker Panels. Genetics.

[13] Heffner E.L., Sorrells M.E., Jannink J.-L. (2009). Genomic Selection for Crop Improvement. Crop Sci..

[14] Heffner E.L., Lorenz A.J., Jannink J.-L., Sorrells M.E. (2010). Plant Breeding with Genomic Selection: Gain per Unit Time and Cost. Crop Sci..

[15] Crossa J., Campos G.D.L., Pérez P., Gianola D., Burgueño J., Araus J.L., Makumbi D., Singh R.P., Dreisigacker S., Yan J. (2010). Prediction of Genetic Values of Quantitative Traits in Plant Breeding Using Pedigree and Molecular Markers. Genetics.

[16] Lorenz A.J., Chao S., Asoro F.G., Heffner E.L., Hayashi T., Iwata H., Smith K.P., Sorrells M.E., Jannink J.-L. (2011). Genomic Selection in Plant Breeding. Advances in Agronomy.

[17] Hayes B.J., Cogan N.O.I., Pembleton L.W., Goddard M.E., Wang J., Spangenberg G.C., Forster J.W. (2013). Prospects for genomic selection in forage plant species. Plant Breed..

[18] Lorenz A.J. (2013). Resource Allocation for Maximizing Prediction Accuracy and Genetic Gain of Genomic Selection in Plant Breeding: A Simulation Experiment. G3 Genes Genomes Genet..

[19] Meuwissen T.H., Hayes B.J., Goddard M.E. (2001). Prediction of total genetic value using genome-wide dense marker maps. Genetics.

[20] Barton N., Etheridge A., Véber A. (2017). The infinitesimal model: Definition, derivation, and implications. Theor. Popul. Biol..

[21] Collard B.C.Y., Mackill D.J. (2008). Marker-assisted selection: an approach for precision plant breeding in the twenty-first century. Phil. Trans. R. Soc. B.

[22] Bernardo R., Yu J. (2007). Prospects for Genomewide Selection for Quantitative Traits in Maize. Crop Sci..

[23] Grenier C., Cao T.-V., Ospina Y., Quintero C., Châtel M.H., Tohme J., Courtois B., Ahmadi N. (2015). Accuracy of Genomic Selection in a Rice Synthetic Population Developed for Recurrent Selection Breeding. PLoS ONE.

[24] Hickey J.M., Chiurugwi T., Mackay I., Powell W., Implementing Genomic Selection in CGIAR Breeding Programs Workshop Participants (2017). Genomic prediction unifies animal and plant breeding programs to form platforms for biological discovery. Nat. Genet..

[25] Morais O.P., Duarte J.B., Breseghello F., Coelho A.S.G., Morais O.P., Magalhães A.M. (2018). Single-Step Reaction Norm Models for Genomic Prediction in Multienvironment Recurrent Selection Trials. Crop Sci..

[26] Müller D., Schopp P., Melchinger A.E. (2017). Persistency of Prediction Accuracy and Genetic Gain in Synthetic Populations under Recurrent Genomic Selection. G3 (Bethesda).

[27] Windhausen V.S., Atlin G.N., Hickey J.M., Crossa J., Jannink J.-L., Sorrells M.E., Raman B., Cairns J.E., Tarekegne A., Semagn K. (2012). Effectiveness of genomic prediction of maize hybrid performance in different breeding populations and environments. G3 (Bethesda).

[28] Weyhrich R.A., Lamkey K.R., Hallauer A.R. (1998). Responses to Seven Methods of Recurrent Selection in the BS11 Maize Population. Crop Sci..

[29] Habyarimana E., Lorenzoni C., Redaelli R., Alfieri M., Amaducci S., Cox S. (2018). Towards a perennial biomass sorghum crop: A comparative investigation of biomass yields and overwintering of Sorghum bicolor x S. halepense lines relative to long term S. bicolor trials in northern Italy. Biomass Bioenergy.

[30] Federer W.T. (1956). Augmented (or hoonuiaku) designs. Hawaii. Plant. Rec..

[31] Alfieri M., Cabassi G., Habyarimana E., Quaranta F., Balconi C., Redaelli R. (2019). Discrimination and prediction of polyphenolic compounds and total antioxidant capacity in sorghum grains. J. Near Infrared Spectrosc..

[32] R Core Team R: A Language and Environment for Statistical Computing. https://www.r-project.org/.

[33] Duane S., Kennedy A.D., Pendleton B.J., Roweth D. (1987). Hybrid Monte Carlo. Phys. Lett. B.

[34] Neal R.M. (2012). MCMC using Hamiltonian dynamics. arXiv.

[35] Hoffman M.D., Gelman A. (2011). The No-U-Turn Sampler: Adaptively Setting Path Lengths in Hamiltonian Monte Carlo. arXiv.

[36] Bürkner P.-C. (2017). Brms: An R Package for Bayesian Multilevel Models Using Stan. J. Stat. Softw..

[37] Piepho H.-P., Möhring J. (2007). Computing Heritability and Selection Response from Unbalanced Plant Breeding Trials. Genetics.

[38] Habyarimana E. (2016). Genomic prediction for yield improvement and safeguarding genetic diversity in CIMMYT spring wheat (Triticum aestivum L.). Aust. J. Crop Sci..

[39] Habyarimana E., Parisi B., Mandolino G. (2017). Genomic prediction for yields, processing and nutritional quality traits in cultivated potato (Solanum tuberosum L.). Plant Breed..

[40] De los Campos G., Gianola D., Rosa G.J.M., Weigel K.A., Crossa J. (2010). Semi-parametric genomic-enabled prediction of genetic values using reproducing kernel Hilbert spaces methods. Genet. Res..

[41] Xu S., Zhu D., Zhang Q. (2014). Predicting hybrid performance in rice using genomic best linear unbiased prediction. Proc. Natl. Acad. Sci. USA.

[42] Danecek P., Auton A., Abecasis G., Albers C.A., Banks E., DePristo M.A., Handsaker R.E., Lunter G., Marth G.T., Sherry S.T. (2011). The variant call format and VCFtools. Bioinformatics.

[43] Pritchard J.K., Stephens M., Donnelly P. (2000). Inference of population structure using multilocus genotype data. Genetics.

[44] Evanno G., Regnaut S., Goudet J. (2005). Detecting the number of clusters of individuals using the software STRUCTURE: A simulation study. Mol. Ecol..

[45] Excoffier L., Smouse P.E., Quattro J.M. (1992). Analysis of molecular variance inferred from metric distances among DNA haplotypes: application to human mitochondrial DNA restriction data. Genetics.

[46] Pérez P., de los Campos G. (2014). Genome-wide regression and prediction with the BGLR statistical package. Genetics.

[47] VanRaden P. (2008). Efficient Methods to Compute Genomic Predictions. J. Dairy Sci..

[48] Gianola D. (2013). Priors in Whole-Genome Regression: The Bayesian Alphabet Returns. Genetics.

[49] Piepho H.P., Möhring J., Melchinger A.E., Büchse A. (2008). BLUP for phenotypic selection in plant breeding and variety testing. Euphytica.

[50] Falconer D.S., Mackay T.F.C. (1996). Introduction to Quantitative Genetics.

[51] De los Campos G., Naya H., Gianola D., Crossa J., Legarra A., Manfredi E., Weigel K., Cotes J.M. (2009). Predicting quantitative traits with regression models for dense molecular markers and pedigree. Genetics.

[52] Clark S.A., Hickey J.M., Daetwyler H.D., van der Werf J.H. (2012). The importance of information on relatives for the prediction of genomic breeding values and the implications for the makeup of reference data sets in livestock breeding schemes. Genet. Sel. Evol..

[53] Scutari M., Mackay I., Balding D. (2016). Using Genetic Distance to Infer the Accuracy of Genomic Prediction. PLoS Genet..

[54] Raschka S. (2018). Model Evaluation, Model Selection, and Algorithm Selection in Machine Learning. arXiv.

[55] Gelman A., Rubin D.B. (1992). Inference from Iterative Simulation Using Multiple Sequences. Statist. Sci..

[56] Wu Y., Li X., Xiang W., Zhu C., Lin Z., Wu Y., Li J., Pandravada S., Ridder D.D., Bai G. (2012). Presence of tannins in sorghum grains is conditioned by different natural alleles of Tannin1. Proc. Natl. Acad. Sci. USA.

[57] Dia V.P., Pangloli P., Jones L., McClure A., Patel A. (2016). Phytochemical concentrations and biological activities of Sorghum bicolor alcoholic extracts. Food Funct..

[58] López-Contreras J.J., Zavala-García F., Urías-Orona V., Martínez-Ávila G.C.G., Rojas R., Niño-Medina G. (2015). Chromatic, Phenolic and Antioxidant Properties of Sorghum bicolor Genotypes. Not. Bot. Horti Agrobo..

[59] Dykes L. (2007). Phenolic Compounds in Cereal Grains and Their Health Benefits. Cereal Food World.

[60] Piper J., Kulakow P. (2011). Seed yield and biomass allocation in Sorghum bicolor and F1 and backcross generations of S.bicolor X S.halepense hybrids. Can. J. Bot..

[61] Paterson A.H. (2008). Genomics of Sorghum. Int. J. Plant Genom..

[62] Kaur R., Soodan A.S. (2017). Reproductive biology of Sorghum halepense (L.) Pers. (Poaceae; Panicoideae; Andropogoneae) in relation to invasibility. Flora.

[63] Cox S., Nabukalu P., Paterson A., Kong W., Nakasagga S. (2018). Development of Perennial Grain Sorghum. Sustainability.

[64] De Donato M., Peters S.O., Mitchell S.E., Hussain T., Imumorin I.G. (2013). Genotyping-by-Sequencing (GBS): A Novel, Efficient and Cost-Effective Genotyping Method for Cattle Using Next-Generation Sequencing. PLoS ONE.

[65] Xu Y., Yang T., Zhou Y., Yin S., Li P., Liu J., Xu S., Yang Z., Xu C. (2018). Genome-Wide Association Mapping of Starch Pasting Properties in Maize Using Single-Locus and Multi-Locus Models. Front. Plant Sci..

[66] Norman A., Taylor J., Edwards J., Kuchel H. (2018). Optimising Genomic Selection in Wheat: Effect of Marker Density, Population Size and Population Structure on Prediction Accuracy. G3.

[67] Heffner E.L., Jannink J.-L., Sorrells M.E. (2011). Genomic Selection Accuracy using Multifamily Prediction Models in a Wheat Breeding Program. Plant Genome.

